# Effectiveness of Extracorporeal Shockwave Therapy on Controlling Spasticity in Cerebral Palsy Patients: A Meta-Analysis of Timing of Outcome Measurement

**DOI:** 10.3390/children10020332

**Published:** 2023-02-09

**Authors:** Min Cheol Chang, You Jin Choo, Sang Gyu Kwak, Kiyeun Nam, Sae Yoon Kim, Hee Jin Lee, Soyoung Kwak

**Affiliations:** 1 Department of Rehabilitation Medicine, College of Medicine, Yeungnam University, Daegu 42415, Republic of Korea; 2 Department of Medical Statistics, College of Medicine, Catholic University of Daegu, Daegu 42472, Republic of Korea; 3 Department of Rehabilitation Medicine, Dongguk University College of Medicine, Goyang 10326, Republic of Korea; 4 Department of Pediatrics, College of Medicine, Yeungnam University, Daegu 42415, Republic of Korea

**Keywords:** cerebral palsy, extracorporeal shockwave therapy, range of motion, plantar surface area, modified ashworth scale, meta-analysis

## Abstract

Extracorporeal shockwave therapy (ESWT) has been suggested as an alternative treatment for reducing spasticity in patients with cerebral palsy (CP). However, the duration of its effect was rarely known. A meta-analysis was performed to investigate the effectiveness of ESWT at controlling spasticity in patients with CP according to the follow-up period. We included studies in which ESWT was used to manage spasticity in patients with CP, and the effect was compared with that in a control group. Finally, three studies were included. In the meta-analysis, spasticity, measured using the modified Ashworth scale (MAS), was significantly reduced after ESWT compared with that in the control group; however, it was sustained for only 1 month. After ESWT, significant increases in passive ankle range of motion (ROM) and plantar surface area in the standing position were observed compared with those in the control group and sustained for up to 3 months. Although spasticity measured using MAS was significantly reduced for only 1 month, improvement in spasticity-associated symptoms, such as ankle ROM and plantar surface area contacting the ground, persisted for over 3 months. ESWT appears to be a useful and effective therapeutic option for managing spasticity in patients with CP.

## 1. Introduction

Cerebral palsy (CP) is a group of disorders that can cause abnormalities in movement, muscle tone, balance, and posture caused by non-progressive disorders of the immature brain [1]. Movement problems are the main feature of CP, but difficulties in learning, thinking, feeling, communication, and behavior are often comorbid. Depending on the brain injury area and severity, disabilities can manifest in various domains, including motor, sensory, balance, cognition, communication, and behavior. In recent studies, the average incidence of CP varies between 1.5 and 3.0 per 1000 live births [1]. Children with CP usually present with coexisting deformities, neural factors (weakness, hypertonia, and impairment of selective motor control), and non-neural factors (contracture of muscle and soft tissue) [2]. In addition, most patients with CP suffer from muscle spasticity, which can cause discomfort and functional disability due to muscle tightness and joint stiffness, thus decreasing their quality of life [3,4]. Spasticity is a component of upper-motor neuron syndrome, which features a velocity-dependent increase in muscle tone or tonic stretch reflexes with exaggerated tendon jerks caused by hyperexcitability of the stretch reflex [4].

Previous studies reported that the severity of muscle spasticity in patients with CP had a significant correlation with the Gross Motor Function Measure [5,6]. Furthermore, Ali found a strong positive correlation between the severity of spasticity in patients with CP and their quality of life [7]. Because of its close relationship with functional ability and quality of life, the appropriate management of spasticity is an important issue for the management of patients with CP.

For the management of spasticity, transcutaneous electrical stimulation, transcranial direct current stimulation, repetitive transcranial magnetic stimulation, acupuncture, cryotherapy, thermotherapy, vibratory stimulation, orthoses, electromyography biofeedback, therapeutic ultrasound, passive movement, and muscle stretching are used in clinical practice [8,9,10,11], but scientific evidence is lacking. Botulinum toxin injection has become widely used to control spasticity in patients with CP, and its effect has been fully demonstrated in several studies [12,13,14]. However, it has disadvantages in that it is expensive and involves pain during injection. Moreover, botulinum toxin injections can cause excessive motor weakness or atrophy of the injected muscle and fatigue [15]. Additionally, after a botulinum injection, neutralizing antibodies are formed in more than 30% of spastic patients and labeled as “non-responders” [16]. Increased intramuscular connective tissue and fat content in injected muscles are other causes of the lack of response to botulinum toxin injection [17]. 

In addition to botulinum toxin, phenol or alcohol can be injected into spastic muscles, but these are limited by severe procedural pain, persistent pain after injection, and occasional nerve damage [18,19]. When spasticity is not effectively controlled by conservative methods and is severe, an orthopedic surgery can be considered the last therapeutic option [20]. However, surgery can cause secondary problems such as muscle shortening, bony deformities, and joint contractures. Therefore, several new treatment modalities for managing spasticity have been proposed. Extracorporeal shockwave therapy (ESWT) in particular has been proposed to effectively reduce muscle spasticity in patients with CP.

ESWT was first used in 1980 to treat kidney stones in urology patients. Shock waves are produced through the rapid propagation of rapidly increased pressure in three-dimensional space, which results in sequences of high-energy biphasic acoustic impulses. Shock waves can be applied by focusing on targeted body tissues without damaging the overall structures [21]. The pain control effect of ESWT was demonstrated in several previous studies. Therefore, in clinical practice, ESWT is being applied for controlling pain from various types of musculoskeletal disorders [22]. Recently, ESWT was reported that it has an effect on controlling spasticity occurred following lesions in the central nervous system. Santamato et al. found that ESWT enhanced the therapeutic effect of botulinum toxin injection in reducing spasticity after stroke, and its effectiveness was greater than that of electrical stimulation [23]. Guo et al. conducted a meta-analysis to evaluate the effects of ESWT on controlling spasticity in patients with stroke and proved its ability to reduce spasticity of the upper and lower extremities [24].

Recently, ESWT has been proposed as an alternative treatment for reducing spasticity in patients with CP [25,26,27,28,29]. ESWT has the advantage that it can be easily and conveniently applied without significant procedural pain and rarely causes considerable complications. Several previous studies have evaluated the effectiveness of ESWT in reducing spasticity in patients with CP [25,26,27,28,29]. In 2019, Kim et al. conducted a meta-analysis and found that ESWT had a positive spasticity reduction effect; however, only effectiveness was assessed immediately after ESWT termination [30]. Therefore, clinicians should be aware of the duration of the spasticity-reducing effect after the completion of ESWT. 

In this study, we investigated the effect of ESWT on spasticity in patients with CP during the follow-up period.

## 2. Materials and Methods

### 2.1. Search Strategy

We performed a meta-analysis following the Preferred Reporting Items for Systematic Reviews and Meta-Analyses guidelines. We registered the protocol for this meta-analysis on the International Platform of Registered Systematic Review and Meta-analysis Protocols (INPLASY protocol no. INPALSY202280066) [31]. We systematically searched the PubMed, Embase, SCOPUS, and Cochrane Library databases for related studies published between 1 January 1980 and 10 August 2022. A list of search terms and strategies (PubMed, Embase, SCOPUS, and Cochrane Library databases) is provided in the published protocol [30]. Studies involving human participants were identified using the filter function of each database. All study designs were considered. Only articles written in English were included. More detailed information regarding search strategy can be found in Supplementary materials File S1.

### 2.2. Eligibility Criteria

We selected studies according to the following criteria: (1) ESWT was conducted for the management of spasticity in patients with CP; (2) the effect of ESWT was evaluated before and after treatment; and (3) the effect of ESWT was evaluated by comparing the results of the experimental and control groups. The exclusion criteria were as follows: (1) anti-spastic medications or botulinum toxin injections were conducted to participants in the control group; (2) the timing of the ESWT effect has not been clarified; (3) previous history of neurosurgery or orthopedic surgery for reducing spasticity; and (4) case reports, review articles, conference presentations or letters, and studies lacking sufficient data or results.

### 2.3. Study Selection and Data Extraction

Duplicate publications were eliminated before two independent reviewers (MCC and YJC) assessed potentially eligible studies for inclusion in the analysis. The screening was based on a review of the titles and abstracts of the articles. Disagreements were resolved through consensus. After the primary screening, the two reviewers (MCC and YJC) independently investigated the full text of the eligible studies. The collected data comprised the first author’s name, publication year, sample size, demographic data, ESWT protocol, outcome variables (modified Ashworth scale [MAS], passive ankle range of motion [ROM], plantar surface area contacting the ground during standing [mm^2^], and adverse effects). Since there was insufficient data in Vidal et al. [29], additional data were obtained by contacting the authors. 

### 2.4. Methodological Quality Assessment 

Two different tools were utilized in the assessment of the methodological quality of the included studies. The Cochrane Collaboration’s Handbook was used for assessing the risk of bias for randomized control trials (RCTs), and the evaluation factors included were: (1) adequate sequence generation, (2) blinding, (3) incomplete outcome data, (4) allocation concealment, (5) selective outcome reporting, and (6) other potential sources of bias. The judgment of bias was expressed as “low risk,” “high risk,” or “unclear risk” [32]. In addition, the Newcastle–Ottawa scale (NOS) was used to determine the quality of the crossover study in three aspects: subject selection, assessment of outcome, and group comparability [32]. The quality of the included studies was graded as low (0–3), moderate (4–6), or high (7–9) [33]. Discrepancies were resolved via consensus.

### 2.5. Statistical Analysis

Review management software (RevMan 5.3) was used for the statistical analysis of the pooled data. For each analysis, a heterogeneity test was performed using I^2^ statistics to measure the degree of inconsistency between the results. When the I^2^ value was less than or equal to 50%, the pooled data were considered homogeneous, and a fixed-effects model was used for analysis. Conversely, if the I^2^ value was greater than 50%, the pooled data were considered significant heterogeneity, and a random-effects model was applied. Continuous variables were analyzed, and standardized mean differences (SMDs) and 95% confidence intervals (CIs) were calculated. Statistical significance was set at *p* < 0.05. A funnel plot and Egger’s test were used to evaluate the publication bias using R version 4.1.2. The publication bias of individual studies was determined based on pooled estimates using funnel plots. We used Egger’s test to determine whether the funnel plot was symmetrical. *p* < 0.05 was set as indicating the possibility of publication bias.

## 3. Results

### 3.1. Study Selection

**Figure 1:**
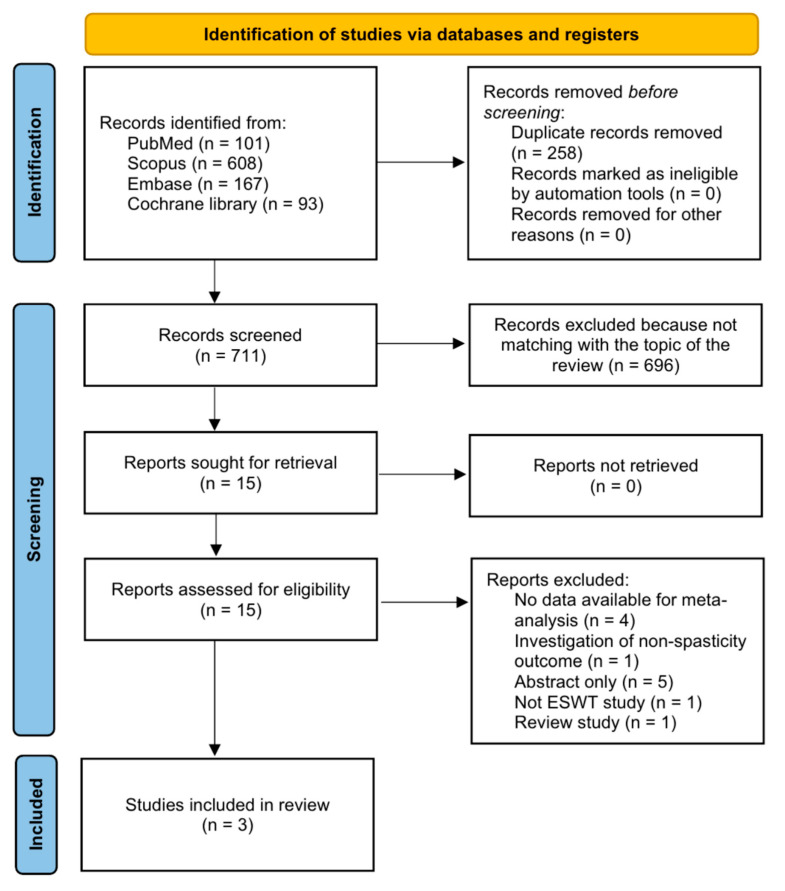
Flowchart showing search result selection.

A preliminary search of all databases resulted in the selection of 969 potentially relevant studies (Figure 1). A total of 258 duplicate studies were excluded, and an additional 696 publications were excluded after reviewing titles and abstracts. The remaining 15 studies were assessed by reviewing the full texts of the articles. After the systematic review, 12 studies were excluded (four studies: no data available for meta-analysis; one study: investigation of non-spasticity outcome; five studies: abstract only; one study: no ESWT study; and one study: review study). Finally, three articles, including one RCT (Vidal et al. [29]) and two crossover studies (Amelio et al. [25] and Gonkova et al. [27]), were included in the final analysis.

### 3.2. Study Characteristics

The selected studies included 47 patients with CP. In a study by Amelio et al. [25], 12 patients received ESWT and placebo ESWT on the gastrocnemius and soleus muscles. Gonkova et al. administered ESWT to 40 plantar flexor muscle groups (gastrocnemius and soleus muscles) in 25 patients [27]. Vidal et al. administered ESWT to 27 spastic muscles from 10 patients after allocation to either the control or experimental group [29]. The characteristics of the included studies are summarized in Table 1. 

### 3.3. Risk of Bias

Vidal et al.’s study was an RCT, and the risk of bias was assessed using Cochrane Handbook 5.1 Assessment Tool. Vidal et al. reported a low risk of bias in random sequence generation [29]. However, their study had high risks of allocation concealment, blinding of participants and personnel, blinding of outcome assessment, incomplete outcome data, selective reporting, and other domains. The other two crossover studies were evaluated using NOS [26,28]. Both studies were scored 8 stars (selection of participants: 3 stars; comparability of groups: 2 stars; outcome assessment: 3 stars), indicating a low risk of bias.

### 3.4. Meta-Analysis Results

**Figure 2:**
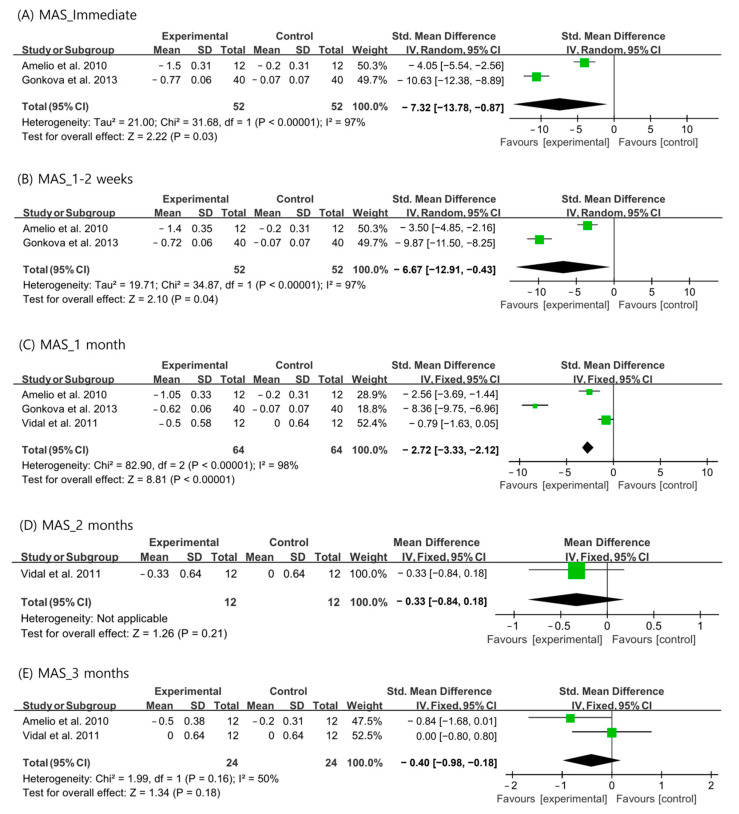
Changes in modified Ashworth scale score after extracorporeal shockwave therapy [25,27,29].

Changes in MAS scores after ESWT were analyzed using data recorded immediately, 1–2 weeks, 1 month, 2 months, and 3 months after ESWT. Immediately, 1–2 weeks, and 1 month after ESWT, the random-effects model was adopted because the I^2^ values were > 50%. For outcomes at 2 and 3 months after ESWT, a fixed-effects model was adopted. Analysis of the changes in MAS revealed a significantly larger reduction than that in the control group immediately, 1–2 weeks, and 1 month after ESWT (immediate: SMD = −7.32, 95% CI = −13.78 to −0.87, *p* = 0.03; 1–2 weeks: SMD = −6.67, 95% CI = −12.91 to −0.43, *p* = 0.04; and 1 month: SMD = −2.27, 95% CI = −3.33 to −2.12, *p* < 0.001) (Figure 2). However, MAS was not significantly reduced after ESWT at the 2- and 3-month follow-ups than that in the control group (2 months: SMD = −0.33, 95% CI = −0.84 to 0.18, *p* = 0.21; 3 months: SMD = −0.40, 95% CI = −0.98 to 0.18, *p* = 0.18) (Figure 2). 

**Figure 3:**
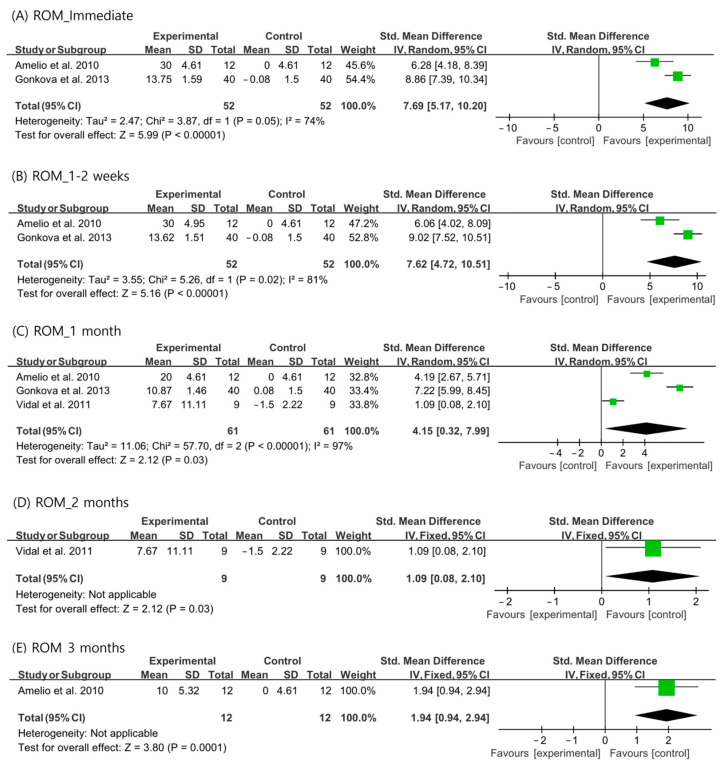
Changes in ankle range of motion after extracorporeal shockwave therapy [25,27,29].

For passive ankle ROM, the random-effects model was used to analyze the immediate, 1–2-week, and 1-month outcomes because the I^2^ values were >50%. A fixed-effects model was used for the analysis of the 2- and 3-month outcomes. The ankle ROMs were significantly more increased than those in the control group at all the evaluation time periods (immediate: SMD = 7.69, 95% CI = 5.17 to 10.20, *p* < 0.001; 1–2 weeks: SMD = 7.62, 95% CI = 4.72 to 10.51, *p* < 0.001; 1 month: SMD = 4.15, 95% CI = 0.32 to 7.99, *p* = 0.03; 2 months: SMD = 1.09, 95% CI = 0.08 to 2.10, *p* = 0.03; and 3 months: SMD = 1.94, 95% CI = 0.94 to 2.94, *p* < 0.001) (Figure 3).

**Figure 4:**
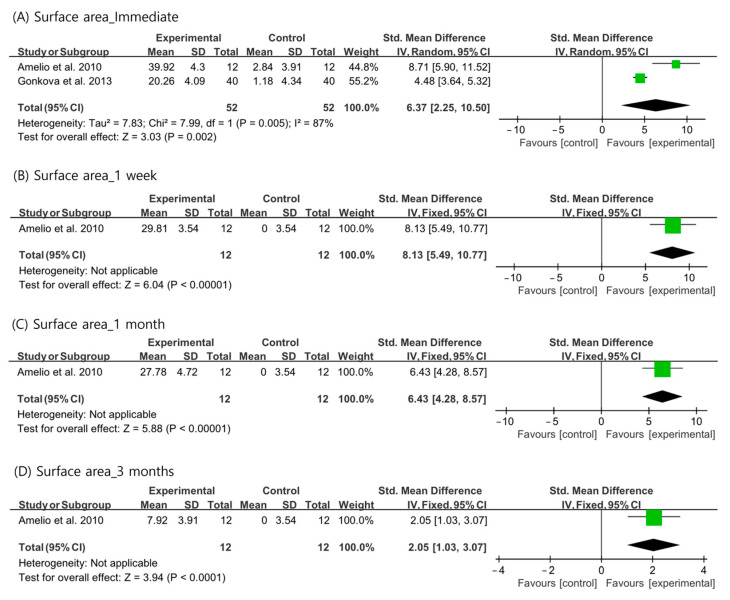
Changes in plantar surface area contacting the ground during standing after extracorporeal shockwave therapy [25,27,29].

Regarding the plantar surface area contacting the ground during standing, the outcomes immediately after ESWT were analyzed using a random-effects model (I^2^ = 87%). Data at 1 week, 1 month, and 3 months after ESWT were analyzed using a fixed-effects model. The plantar surface area contacting the ground during standing was significantly increased after ESWT at all evaluation time periods than that in the control group (immediate: SMD = 6.37, 95% CI = 2.25 to 10.50, *p* = 0.002; 1 week: SMD = 8.13, 95% CI = 5.49 to 10.77, *p* < 0.001; 1 month: SMD = 6.43, 95% CI = 4.28 to 8.57, *p* < 0.001; and 3 months: SMD = 2.05, 95% CI = 1.03 to 3.07, *p* < 0.001) (Figure 4).

Furthermore, Amelio et al. and Gonkova et al. reported no adverse effects. However, in the study by Vidal et al., small superficial hematomas, petechiae, and light pain occurred during ESWT in three patients.

### 3.5. Publication Bias

**Figure 5:**
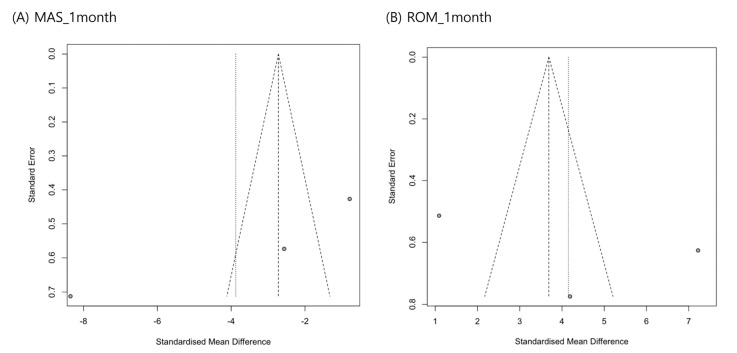
Graphic funnel plots of differences in modified Ashworth scale scores (**A**) and ankle range of motion changes (**B**) at 1 month after extracorporeal shockwave therapy.

A funnel plot and Egger’s test were used to assess publication bias for MAS and ankle ROM changes 1 month after ESWT. The funnel plots appeared symmetrical, and Egger’s test revealed no significant publication bias (MAS score at 1 month, *p* = 0.211; ROM at 1 month, *p* = 0.614) (Figure 5).

## 4. Discussion

In our meta-analysis, spasticity measured using MAS was significantly reduced by a greater degree after ESWT than in the control group. However, the reduction in spasticity was sustained for 1 month and undetectable at 2 and 3 months after ESWT. However, after ESWT, significant increases in passive ankle ROM and plantar surface area contacting the ground during standing were found compared with that in the control group. Their increments were sustained for up to 3 months after ESWT. No major adverse effects were reported in any of the included studies.

Moreover, when a significant change existed in spasticity, ankle ROM, and plantar surface area contacting the ground during standing, the absolute value of the effect size ranged from 1.94 to 8.13. Based on Cohen’s study, these effect size values are interpreted to significantly reduce spasticity or spasticity-associated symptoms in patients with CP [34].

The pathophysiology of spasticity or hypertonus is complex and not fully understood. It is caused by various spinal and supraspinal pathways as well as multiple neuronal mechanisms that lead to increased reflex excitability. Recent studies have revealed more regarding the pathophysiology of spasticity. Downregulation of the potassium chloride cotransporter, KCC2, in motor neuron membranes appears to be involved in experimentally induced central nervous system injury in rodents [35]. This depolarization depolarizes the chloride equilibrium potential and weakens postsynaptic inhibition [35]. After injury of the central nervous system, it appears to be countered by brain-derived neurotrophic factors. Spasticity can cause pain, interfere with function, and disrupt sleep in most children with CP.

El-Shamy et al. conducted a study to evaluate the effects of ESWT on gait pattern in patients with hemiplegic CP and found that several gait parameters, including cadence, speed, stride length, stance phase time, and cycle time, improved significantly after the application of ESWT [26]. Amelio et al. and Gonkova et al. used baropodometric outcomes such as plantar surface area and peak pressure values to measure the effects of ESWT [25,27]. Baropodometric outcomes showed a significant increase in foot contact area during gait, but this increase was not observed in the peak pressure under the heel immediately after the application of ESWT. Although not statistically significant, heel peak pressure during gait improved following ESWT. The results of these previous studies have shown that ESWT can decrease hypertonia in foot flexors treated with ESWT and can change the postural attitude and body stability of patients with CP. Reduction in plantar flexor hypertonia indicates an increase in the entire plantar surface area on the affected side. This effect was comparable to that of botulinum toxin injections into the muscles [25]. 

The mechanism by which ESWT controls spasticity has not been elucidated. However, it has been proposed that ESWT can cause rheological effects, which alter the elasticity and extensibility of the muscles [36,37]. ESWT can also reportedly induce nitric oxide production to function on neuromuscular junctions and modulate the secretion of interleukins [38,39]. ESWT might induce a cascade of interactions between physical shockwave energy and biological responses, such as the expression of angiogenesis-related growth factors, including proliferating cell nuclear antigen, vessel endothelial growth factor, endothelial nitric oxide synthase, and neovascularization [40,41]. Furthermore, the application of ESWT to spastic muscles reportedly induces transient nerve conduction dysfunction at neuromuscular junctions [42]. Accordingly, ESWT seems to work at the muscle and neuromuscular junction level but not that of the nervous system. Abnormal muscle tightness or hypertonus due to spasticity develops because of the enhanced activation of α and γ motor neurons after upper motor neuron lesions occur [43]. Therefore, the development of spasticity can be ascribed to pathological alterations in the muscle and nervous system. The non-action of ESWT in the nervous system is likely related to our finding that the decrease in MAS score persisted for only 1 month. 

In clinical practice, two types of shockwave generators are used: focused ESWT and radial ESWT. These two types of ESWT differ in physical properties, generation modes, magnitudes of standard parameters applied, and depths attained. Electromagnetic, electrohydraulic, and piezoelectric sources are used to produce focused ESWT. The pressure produced by the focused ESWT rapidly increases, and energy is absorbed as deep as 12 cm [21]. Due to the relatively low energy dispensed, it might cause less damage to the skin and underlying soft tissue. A pneumatic system is used to generate radial ESWT. Most of the energy is concentrated at the probe tip and transmitted radially into the tissue [44]. The pressure produced by radial ESWT increases much more slowly than that produced by focused ESWT, and the depth of energy is shallow at only 3–4 cm. Focused ESWT is generally more intense within a target area, whereas radial ESWT acts on a more widespread but superficial region [21]. Accordingly, radical ESWT is considered less invasive than focused ESWT and more suitable for physiotherapy [45]. Despite the above knowledge, conclusive evidence is lacking regarding which ESWT type is more effective at managing spasticity.

Consensus is lacking on the appropriate or most effective number of shockwave impulses and sessions. However, 2000–3000 shockwaves and strike frequencies of 1–8 Hz applied at weekly intervals are recommended [46]. Bleeding disorders and pregnancy are contraindications for ESWT. Most previous studies reported no major complications of ESWT. Most adverse effects after ESWT were minor, including redness of the skin, pain in application areas, skin erosion, transient bone edema, and soft-tissue swelling [47,48]. 

In 2019, Kim et al. [30] conducted a meta-analysis that included five previous studies to evaluate the effect of ESWT in reducing spasticity in patients [25,26,27,28,29]. However, they evaluated only the immediate effects of ESWT. In addition, in one (Picelli et al. [28]) of the five included studies [25,26,27,28,29], botulinum toxin injection was administered to patients in the control group, and Kim et al. used data obtained from the control group of Picelli et al.’s study for their meta-analysis. Because botulinum toxin injection has a significant spasticity-reducing effect, it should not have been included as a control group in the meta-analysis by Kim et al. Moreover, another study (El-Shamy et al. [26]) included in Kim et al.’s meta-analysis did not clearly describe the evaluation time points. They administered single sessions of ESWT at 1-week intervals for 3 months. Although they claimed to have assessed spasticity at the end of 3 months of treatment, the measurement timing was not described in a manner that readers could easily understand. Therefore, in our meta-analysis, we excluded the studies by Picelli et al. and El-Shamy et al. [26,28]. 

Our meta-analysis was limited because we included only a small number of previous studies. Furthermore, because of the insufficient number of previous studies, we could not perform the analysis according to the shockwave type or application mode. Moreover, the designs of the studies examined in our meta-analysis differed considerably. To obtain more accurate knowledge of ESWT on spasticity in patients with CP, a larger number of well-designed, high-quality studies should be conducted on this topic. In addition, we analyzed only the MAS to measure the degree of spasticity. MAS is the most commonly used tool for assessing spasticity, and alternatives for measuring changes in spasticity after ESWT are lacking. However, although MAS is widely used to measure spasticity, it cannot describe the complex mechanisms of muscle stiffness. Also, MAS is subjective and depends on the experience of physicians who check for spasticity. Furthermore, it is also affected by soft tissue contracture or tightness and not only by spasticity. In future studies, additional tools are needed to evaluate the degree of spasticity. In addition, studies with combined treatment models are necessary to evaluate the effects of combined ESWT and pharmacological interventions.

## 5. Conclusions

A significantly greater reduction in spasticity, ankle ROM, and plantar surface area contacting the ground during standing was observed in the ESWT versus the control group. While the increase in ankle ROM and plantar surface area contacting the ground during standing persisted for >3 months after ESWT, the reduction in spasticity measured using MAS persisted until 1 month after ESWT. These findings suggest that ESWT is a useful and effective therapeutic option for managing spasticity in patients with CP. To the best of our knowledge, this is the first meta-analysis to investigate the ability of ESWT to reduce spasticity or spasticity-associated symptoms in patients with CP according to the follow-up period. 

## Figures and Tables

**Table 1:** Summary of study characteristics of the included studies.

First Author	Publication Year	Design	Patients	Intervention	Evaluation Time Periods	Outcome Measures	Adverse Effects
Amelio [25]	2010	Crossover study	N = 12 (M:F = 6:6), age (mean ± SD) = 8 ± 2.31 years (range: 6–11)	Experimental group: Focused ESWT: 0.03 mJ/mm^2^, 1500 shots for each gastrocnemius muscle and soleus muscle (mainly in the middle of the belly), 1 session Control group: Placebo ESWT, 1 session	Pre-treatment, immediate, 1 week, 1 month, and 3 months after ESWT	MAS, ROM, and plantar surface area during standing position	No
Gonkova [27]	2013	Crossover study	N = 25 (M:F = 16:9), age (mean ± SD) = 4.84 ± 3.11 years	Experimental group: radial ESWT: 0.075 mJ/mm^2^, 1500 shots for each gastrocnemius muscle and soleus muscle (mainly in the middle of the belly), 5 Hz, 1 session Control group: Placebo ESWT, 1 session	Pre-treatment, immediate, 2 weeks, and 1 month after ESWT	MAS, ROM, and plantar surface area during standing position	No
Vidal [29]	2011	RCT	N = 10 (M:F = 8:2), age (mean) = 31 (range: 10–46)	Experimental group: radial ESWT: 0.010 mJ/mm^2^, 2000 shots for each treated muscle (40 muscles, 6 biceps brachii, 6 wrist flexors, 5 hip adductors, 10 gastrocnemii, and 3 hamstrings), 8 Hz, 3 sessions (1 per a week) Control group: Placebo ESWT, 3 sessions (1 per week)	Pre-treatment, 1, 2, and 3 months after ESWT	MAS, ROM	Small superficial hematoma, petechiae, and light pain during ESWT (three patients)

ESWT, extracorporeal shockwave therapy; MAS, modified Ashworth scale; RCT, randomized controlled trial; ROM, range of motion; SD, standard deviation

## Data Availability

Data analyzed in this study were re-analyzed from existing data.

## Supplementary Materials

The following supporting information can be downloaded at: https://www.mdpi.com/article/10.3390/children10020332/s1, File S1: Search strategy.
