# Supplementary material for: Effectiveness of Extracorporeal Shockwave Therapy on Controlling Spasticity in Cerebral Palsy Patients: A Meta-Analysis of Timing of Outcome Measurement
Source: Children (Basel). 2023 Feb 9;10(2):332. doi: 10.3390/children10020332 (PMC9954893; doi:10.3390/children10020332)
Supplement: Supplementary file 1 [file children-10-00332-s001.zip › children-2112685-supplementary.pdf]

Supplementary 1. Search strategy

A search strategy was developed to identify studies comparing the modified Ashworth scale scores, passive ankle range of motion, and plantar surface area in the standing position between extracorporeal shockwave therapy (ESWT) and placebo ESWT for managing spasticity in patients with cerebral palsy.

The search keywords were combined as follows:

"cerebral palsy", "cerebral palsies", "spastic diplegia", "spastic diplegias", "spasticity", "spastic", "muscle hypertonia"

AND

"shock wave", "shock waves", "extracorporeal shockwave therapy", "ESWT"

The search keywords were devised using a combination of subject indexing terms in the titles and abstracts. For the index related to cerebral palsy, [National Guideline Alliance (UK). Cerebral palsy in under 25s: assessment and management. London: National Institute for Health and Care Excellence (NICE); 2017 Jan. (NICE Guideline, No. 62.) Appendix E, Search strategies.] was referenced. For the index related to shock wave, the previous study, [Ebrahim S, Mollon B, Bance S, Busse JW, Bhandari M. Low-intensity pulsed ultrasonography versus electrical stimulation for fracture healing: a systematic review and network meta-analysis. Can J Surg. 2014 Jun;57(3):E105-18. Appendix 1. Search strategies.] was referenced.

Search strategy for: National Guideline Alliance (UK). Cerebral palsy in under 25s: assessment

and management. London: National Institute for Health and Care Excellence (NICE); 2017 Jan. (NICE Guideline, No. 62.) Appendix E, Search strategies.

**E.5.6. Database: Embase**	
#	Searches
1	exp ADOLESCENT/
2	(adolescen$ or teen$ or youth$ or young or juvenile? or minors or highschool$).ti,ab,jx.
3	exp CHILD/
4	(child$ or schoolchild$ or “school age” or “school aged” or preschool$ or toddler$ or kid? or kindergar$ or boy? or girl?).ti,ab,jx.
5	exp INFANT/
6	(infan$ or neonat$ or newborn$ or baby or babies).ti,ab,jx.
7	exp PEDIATRICS/ or exp PUBERTY/
8	(p?ediatric$ or pubert$ or prepubert$ or pubescen$ or prepubescen$).ti,ab,jx,ec.
9	or/1-8
10	CEREBRAL PALSY/
11	((cerebral or brain or central) adj2 (pals$ or paralys#s or pares#s)).ti,ab.
12	little? disease.ti,ab.
13	or/10-12
14	exp NUCLEAR MAGNETIC RESONANCE IMAGING/
15	(MRI or NMRI).ti,ab.
16	((magnetic resonance or MR or MTC or MT or NMR or magneti#ation transfer or spin or chemical shift or diffus$ tensor) adj2 (imag$ or tomogra$)).ti,ab.
17	or/14-16
18	*MUSCLE HYPERTONIA/ or *SPASTICITY/ or *SPASTIC PARAPLEGIA/ or *SPASTIC PA-RESIS/ or *MUSCLE HYPOTONIA/
19	*DYSKINESIA/ or *ATAXIA/ or *ATAXIC GAIT/ or *CEREBELLAR ATAXIA/ or *ATHETOSIS/ or *CHOREA/ or *CHOREOATHETOSIS/ or *DYSTONIA/ or *HYPERKINESIA/ or *HYPOKINESIA/
20	*PARESIS/ or *HEMIPARESIS/ or *HEMIPLEGIA/ or *PARAPLEGIA/ or *QUADRIPLEGIA/
21	(spastic$ or ataxi$ or dyskin$ or dyston$ or chorea or choreoatheto$ or atheto$ or hyper-ton$ or hypoton$ or hyperkin$ or hypokin$ or hyperreflex$ or hemip$ or parap$ or diparesis or diplegia? or quadriparesis or quadriplegia?).ti.
22	or/18-21
23	*BRAIN DISEASE/
24	*ENCEPHALOMALACIA/ or *LEUKOMALACIA/
25	*BRAIN INFARCTION/
26	*HYPOXIC ISCHEMIC ENCEPHALOPATHY/
27	exp *BRAIN MALFORMATION/
28	((brain or cerebral or cerebellum or neuro$ or cortex or cortical or subcortical or encepha-lopath$ or grey matter or white matter or basal ganglia or watershed) adj (pathol$ or mal-form$ or maldevelop$ or injur$ or insult$ or damag$ or lesion? or hypoxi$ or isch?emi$ or an-oxi$ or infarct$ or mutat$)).ti,ab.
29	(periventricular leu#omalacia? or cerebral leu#omalacia? or periventricular encephaloma-lacia? or PVL or porencephal$).ti,ab.
30	or/23-29
31	“SENSITIVITY AND SPECIFICITY”/
32	(sensitivity or specificity).ti,ab.
33	((pre test or pretest or post test or posttest) adj probability).ti,ab.
34	(predictive value$ or PPV or NPV).ti,ab.
35	likelihood ratio$.ti,ab.
36	STATISTICAL MODEL/
37	(ROC curve$ or AUC).ti,ab.
38	diagnos$.ti.
39	(diagnos* adj2 (performance* or accurac* or utilit* or value* or efficien* or effective-ness)).ti,ab.

40	gold standard.ab.
41	*DIAGNOSTIC ACCURACY/ or DIAGNOSTIC TEST ACCURACY STUDY/
42	di.fs.
43	or/31-42
44	predict.ti.
45	(validat* or rule*).ti,ab.
46	(predict* and (outcome* or risk* or model*)).ti,ab.
47	((history or variable* or criteria or scor* or characteristic* or finding* or factor*) and (pre-dict* or model* or decision* or identif* or prognos*)).ti,ab.
48	decision*.ti,ab. and STATISTICAL MODEL/
49	(decision* and (model* or clinical*)).ti,ab.
50	(prognostic and (history or variable* or criteria or scor* or characteristic* or finding* or factor* or model*)).ti,ab.
51	(stratification or discrimination or discriminate or c statistic or "area under the curve" or AUC or calibration or indices or algorithm or multivariable).ti,ab.
52	RECEIVER OPERATING CHARACTERISTIC/
53	or/44-52
54	exp ETIOLOGY/
55	et.fs. [Etiology]
56	(etio$ or aetio$ or cause? or causative or origin? or pathogen$).ti,ab.
57	or/54-56
58	13 and 17 and (30 or 43 or 53 or 57)
59	9 and 17 and 22 and 30 and (43 or 53 or 57)
60	or/58-59
61	limit 60 to english language
62	letter.pt. or LETTER/
63	note.pt.
64	editorial.pt.
65	CASE REPORT/ or CASE STUDY/
66	(letter or comment*).ti.
67	or/62-66
68	RANDOMIZED CONTROLLED TRIAL/ or random*.ti,ab.
69	67 not 68
70	ANIMAL/ not HUMAN/
71	NONHUMAN/
72	exp ANIMAL EXPERIMENT/
73	exp EXPERIMENTAL ANIMAL/
74	ANIMAL MODEL/
75	exp RODENT/
76	(rat or rats or mouse or mice).ti.
77	or/69-76
78	61 not 77

Search strategy for: Ebrahim S, Mollon B, Bance S, Busse JW, Bhandari M. Low-intensity pulsed ultrasonography versus electrical stimulation for fracture healing: a systematic review and network meta-analysis. Can J Surg. 2014 Jun;57(3):E105-18.

**MEDLINE**

1 randomized controlled trial.pt.

2 controlled clinical trial.pt.

3 randomized.ab.

4 placebo.ab.

5 drug therapy.fs.

6 randomly.ab.

7 trial.ab.

8 groups.ab.

9 or/1-8

10 exp animals/ not humans.sh.

11 9 not 10

12 Ultrasonics/

13 Ultrasonic Therapy/

14 High-Energy Shock Waves/

15 (ultraso$ or LIPUS or HIPUS or shock wave$ or shockwave$ or ESWT).tw.

16 or/12-15

17 exp Fractures, Bone/

18 Fracture Healing/

19 exp Bone Remodeling/

20 Bony Callus/

21 fractur$.tw.

22 or/17-21

23 16 and 22

24 (dental or tooth or oral).mp.

25 23 not 24

26 11 and 25

27 limit 26 to yr="2011 - 2012"

28 limit 26 to ed=20111101-20120420

29 27 or 28

Embase

1 clinical trial/

2 randomized controlled trial/

3 randomization/

4 single blind procedure/

5 double blind procedure/

6 crossover procedure/

7 placebo/

8 Randomi?ed controlled trial$.tw.

9 Rct.tw.

10 Random allocation.tw.

11 Randomly allocated.tw.

12 Allocated randomly.tw.

13 (allocated adj2 random).tw.

14 Single blind$.tw.

15 Double blind$.tw.

16 ((treble or triple) adj blind$).tw.

17 Placebo$.tw.

18 prospective study/

19 or/1-18

20 case study/

21 Case report.tw.

22 Abstract Report/ or Letter/

23 or/20-22

24 19 not 23

25 limit 24 to human

26 ultrasound/

27 ultrasound therapy/ or extracorporeal lithotripsy/

28 Low Intensity Pulsed Ultrasound.mp.

29 (ultraso$ or LIPUS or shock wave$ or shockwave$ or ESWT).tw.

30 or/26-29

31 exp fracture/

32 fracture treatment/

33 bone remodeling/

34 fractur$.tw.

35 or/31-34

36 30 and 35

37 (dental or tooth or oral).mp.
38 36 not 37
39 25 and 38
40 limit 39 to yr="2011 - 2012"
41 limit 39 to em=201145-201215
42 40 or 41

Selection of eligible studies

We searched trial registers using the "cerebral palsy", "cerebral palsies", "spastic diplegia", "spastic diplegias", "spasticity", "spastic", "muscle hypertonia", "shock wave", "shock waves", "extracorporeal shockwave therapy", and "ESWT" index, and databases including PubMed, Scopus, Embase, and the Cochrane library for studies published up to August 17, 2022. The results of the database searches were entered into an EndNote X9 library. Duplicates were deleted using the deduplication function in EndNote X9. Two reviewers preferentially removed irrelevant records for selection based on the titles and abstracts. Thereafter, the reviewers checked the full texts to finally select the papers that meet the selection criteria.

Each database was searched under the following conditions:

1. PubMed (101)

   - no filters

2. Scopus (608)

   - no filters

3. Embase (167)

- no filters

4. Cochrane library (93)

- trials

Eligibility criteria

**Population**	Children with cerebral palsy who were managed for spasticity with ESWT were included.
**Intervention**	A paper evaluating the results of ESWT with placebo ESWT was included. We investigated the modified Ashworth scale scores, passive ankle range of motion, and plantar surface area in the standing position.
**Comparison**	Papers comparing the ESWT with placebo ESWT for cerebral palsy with spasticity were included.
**Outcome**	Studies were considered eligible for inclusion in this review if they reported on modified Ashworth scale scores, passive ankle range of motion, and plantar surface area in the standing position.
**Study design**	The design of the studies to be included in this study was not specific and a full range of designs was considered. Consequently, one randomized controlled trial and two crossover design studies were included. Studies published as case reports, reviews, letters, or other undistinctive forms were excluded.
**Limitation**	Publications in a language other than English were excluded.
